# Does Neuroglobin Protect Against Stroke? Insights Into the Role of Neurovascular Unit Cells

**DOI:** 10.1007/s10571-025-01656-9

**Published:** 2026-01-08

**Authors:** María Ángeles Peinado, Santos Blanco, Angela Naranjo, María del Mar Muñoz, Eva Siles, Raquel Hernández, Sara Gröhn, Alejandra Sierra, Esther Martínez-Lara

**Affiliations:** 1 Department of Experimental Biology, University of Jaén, Jaén, Spain; 2 A.I. Virtanen Institute for Molecular Sciences, University of Eastern Finland, Kuopio, Finland

**Keywords:** Neuroglobin therapy, Stroke model, Neurovascular unit, Neuroprotection, Glial activation, Nanoparticles

## Abstract

Ischemic stroke, a leading cause of disability and mortality, initiates a complex damage cascade within the neurovascular unit (NVU), leading to blood-brain barrier (BBB) disruption and neuroinflammation that severely exacerbates secondary injury. Neuroglobin (Ngb), an endogenous protein induced by brain injury, represents a high-potential neuroprotective target. While the precise mechanisms underlying its protective action remain incompletely elucidated, substantial evidence points to its multifaceted ability to mitigate ischemic damage. To fully unlock this potential, a fundamental understanding of how neurons, astrocytes, microglia, and pericytes, coordinate their function in response to stress, and specifically identifying the role Ngb plays within this integrated cellular network, is required. This review examines the post-stroke interplay among these cells, analyzing current knowledge about how Ngb modulates the collective inflammatory response by suppressing pro-inflammatory pathways and fostering a neuroprotective environment. Furthermore, Ngb’s upregulation in glial cells and pericytes promotes direct neuronal repair mechanisms, such as neurite outgrowth and axonal regeneration, while supporting neuronal survival and BBB integrity. Importantly, evidence suggests that Ngb’s efficacy is most pronounced when its intracellular concentration exceeds the levels achieved through physiological upregulation. In this regard, we integrate broad preclinical evidence with specific insights from nanoparticle-mediated delivery systems that enable effective Ngb transport to NVU cells. These synthesized findings demonstrate beneficial outcomes in stroke models, driven by the modulation of mitochondrial dynamics, cytoskeletal remodeling, and synaptic regeneration pathways. Collectively, the literature indicates that targeted therapeutic Ngb may enhancement strategies effectively complement endogenous levels to orchestrate protective responses across the NVU. Nonetheless, a detailed investigation into the therapeutic utility of Ngb is still required to fully translate encouraging preclinical findings into successful clinical application for improving stroke outcomes.

**Graphical Abstract:**

**Figure:**
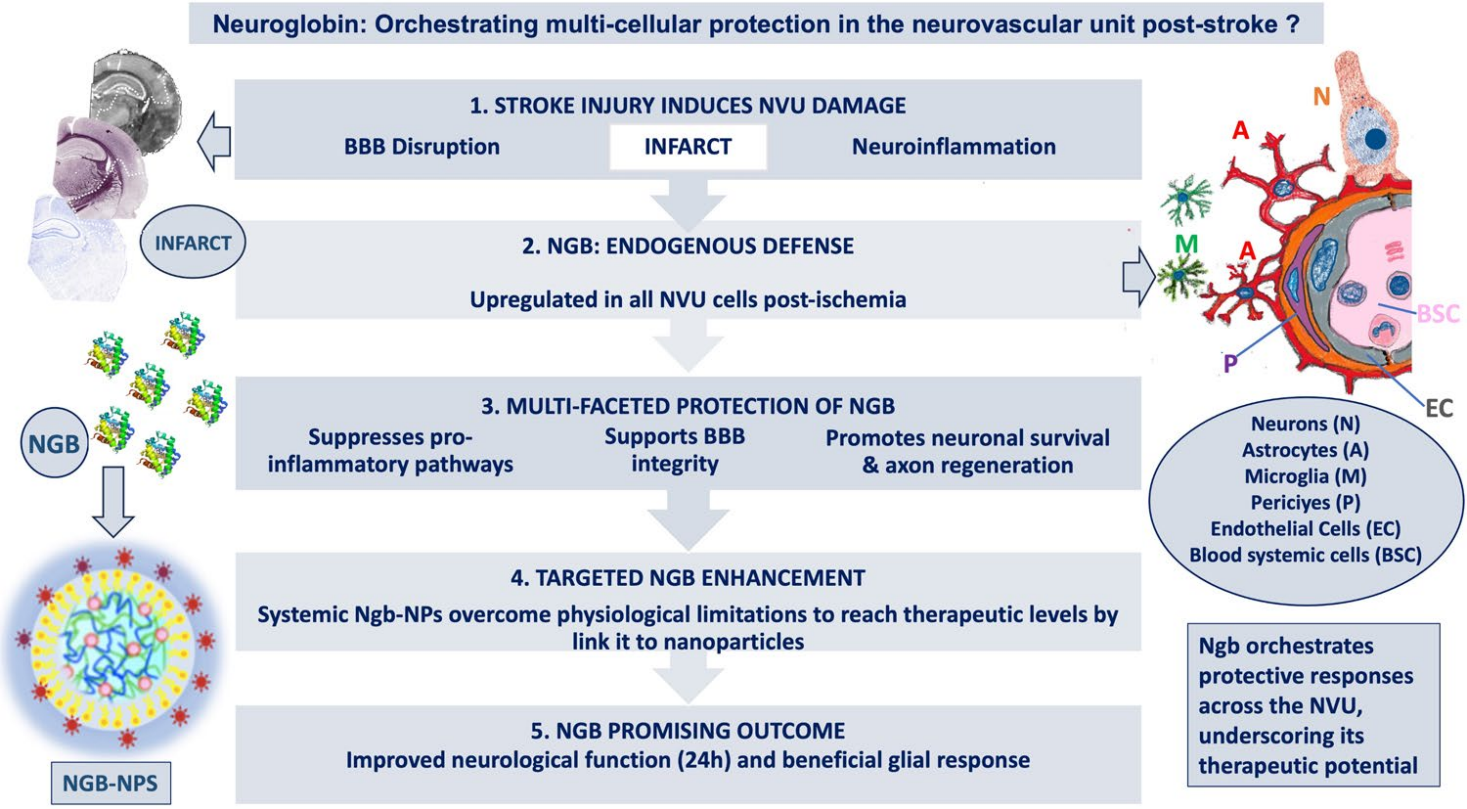
Neuroglobin (Ngb) is an endogenous protein protecting the Neurovascular Unit (NVU) after stroke. This review integrates evidence showing Ngb’s action in glial cells, pericytes, and neurons to suppress inflammation and enhance tissue repair. Systemic Ngb-nanoparticles complement endogenous levels, leading to improved neurological outcomes and underscoring Ngb’s high therapeutic potential

## Introduction

Ischemic stroke, a leading cause of death and neurological impairment, occurs when cerebral blood flow is reduced or interrupted (Zhao et al. 2022). This rapid deprivation of oxygen and nutrients leads to cellular damage, known as the infarct core, and initiates a complex signaling cascade for tissue repair (Xu et al. 2020). The outcome is determined by the interplay of neuronal death, glial activation, and blood-brain barrier (BBB) disruption, all unfolding within the neurovascular unit (NVU). The NVU, a complex multicellular structure, coordinates cerebral blood flow, BBB maintenance, and neuronal function, orchestrating a synchronized response post-stroke (Candelario-Jalil et al. 2022).

**Fig. 1:**
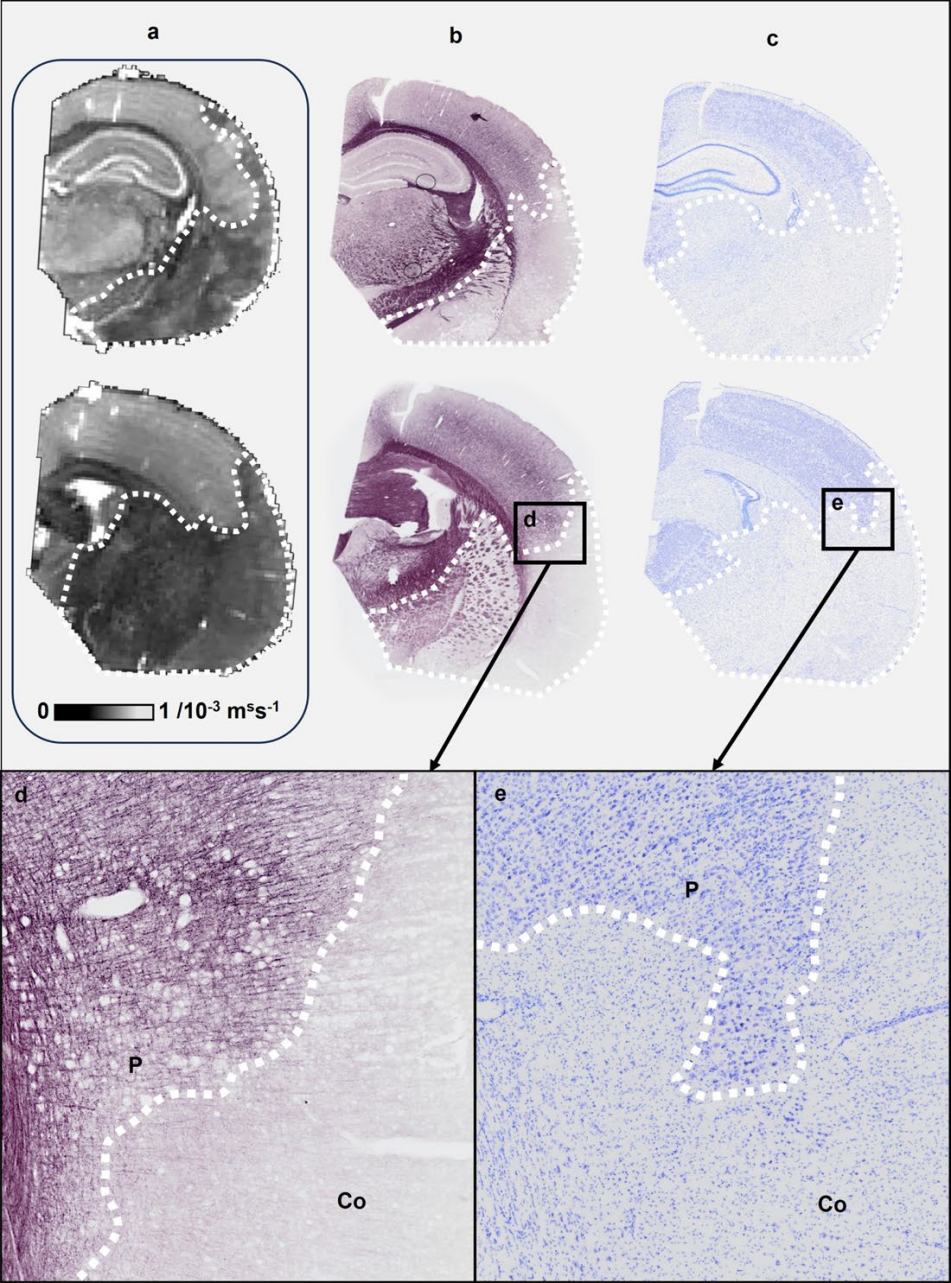
Infarct Visualization. Infarcts from a rat model of tMCAO, 24 h post-ischemia. **a** Conventional T2-weighted MRI, **b** Myelin staining, and **c** Nissl staining are used to visualize and delineate the infarct boundary (white dotted line). Panels (**d**) and (**e**) show magnified views illustrating the distinct boundary between the infarct core (Co) and penumbra (P). Methodological procedures are described in Wang et al. 2008a); Hakkarainen et al. (2016)

Stroke damage evolves in sequential stages: acute (first 72 h), subacute (up to 6 weeks), and chronic (from 6 weeks onwards) (Yu et al. 2024), with a spatiotemporally heterogeneous ischemic response at each stage. This response, highly conditioned by the development of the acute stage, features an infarct core (irreversible damage) and a surrounding penumbra (recovery potential) (Fig. 1). Central to this response is reactive gliosis, a critical component that modulates the immune environment and demarcates the infarct core from the penumbra (Radak et al. 2017), influencing the balance between tissue damage and repair (Li et al. 2023).

Following ischemia, prompt medical intervention is crucial, and immediate therapies are essential to mitigate secondary reperfusion injury (Jia et al. 2024). This damage involves oxidative stress, inflammation, and excitotoxicity, significantly hindering recovery. Therefore, the persistent need for novel therapeutic strategies is critical.

The hemeprotein Neuroglobin (Ngb) has emerged as a key neuroprotective target. Ngb enhances oxygen availability, scavenges reactive species, and modulates inflammatory and cell death pathways, thereby mitigating both primary and reperfusion injury, but its precise protective mechanisms remain incompletely understood (Li et al. 2023). Preclinical evidence strongly supports Ngb neuroprotective capability against stroke (Khan et al. 2006; Wang et al. 2008b), but its efficacy is not uniform across all contexts. This is evidenced by conflicting results in knockout studies and mixed outcomes in exogenous delivery trials, suggesting that factors like the animal model, strain, ischemia type (transient versus permanent), and timing are critical modulators of its therapeutic effect (Luyckx et al. 2019). Consequently, the specific interaction between Ngb and the distinct cellular components of the NVU appears to be a pivotal factor in this protective action, underscoring the importance of targeting the NVU as an integrated system for effective therapeutic intervention (Li et al. 2023).

Given the immense potential and current limitations in stroke therapy, a comprehensive understanding of Ngb’s action in the NVU context is timely and warranted. This review examines the role of Ngb in ischemic stroke within the NVU. Integrating existing literature with preclinical research from the authors’ lab on Ngb-based therapies (Peralta et al. 2019; Blanco et al. 2020, 2022; Peinado et al. 2021), highlighting the therapeutic potential of targeted Ngb delivery. The narrative is supported by high-quality MRI and histological images provided strictly as didactic illustrations serving purely as visual aids to reinforce established findings and concepts fully referenced in the text. For complete methodological details regarding the acquisition of these illustrative images, the reader is directed to the corresponding publications appearing in the figure captions.

## Neuroglobin and Ischemic Stroke

Ngb is a 17 kDa, 150-amino acid, oxygen-binding hemeprotein (Li et al. 2023; Exertier et al. 2022). Discovered in 2000 (Burmester et al. 2000), it is primarily expressed in the nervous system (Brunori and Vallone 2006). Different studies support the neuroprotective capability of Ngb against stroke (Baez et al. 2016; Wen et al. 2018). It has been reported that Ngb exhibits a physical and functional link with mitochondria, supporting its role as a protective respiratory protein (Keppner et al. 2020). Moreover, beyond its gas sensing capability, Ngb acts as a stress-responsive sensor, influencing mechanisms and signaling pathways involved in maintaining mitochondrial function and modulating the neuroinflammatory response (Yu et al. 2012; Peinado et al. 2021; Li et al. 2023). This inherent protective capacity is further substantiated by its rapid transcriptional upregulation in response to cerebral ischemia, which is a critical initial step in harnessing its neuroprotective function (Yu et al. 2012). Nevertheless, the characterization of Ngb neuroprotection is complex; not only do the precise underlying mechanisms remain partially elucidated, but the field also presents heterogeneous data regarding its effects. Specifically, studies utilizing knockout models have generally demonstrated increased vulnerability to ischemic injury following Ngb depletion (Sun et al. 2001; Gøtzsche et al. 2022), and transgenic models exhibiting Ngb overexpression typically display reduced infarct damage (Khan et al. 2006; Wang et al. 2008b; Raida et al. 2013). However, it has been pointed out that care must be exercised when comparing results from different mouse strains and colonies due to the significant influence of the genetic background on the observed phenotype (Raida et al. 2013).

On the other hand, results regarding the exogenous delivery of Ngb protein have been even more complex. While it has been shown to confer protection in diverse models, discrepancies exist. For instance, Cai and collaborators (Cai, et al., 2011) demonstrated that an exogenous Ngb fusion protein containing the TAT transduction domain could efficiently protect the mouse brain from mild or moderate ischemic injury. This contrasts with earlier findings by Peroni et al. (2007), who reported that treatment with the TAT-Ngb fusion protein did not show any significant effect on cell viability in in vitro ischemia models. Notably, studies from our group using systemic Ngb linked to nanoparticles (Ngb-NPs) indicated lower mortality rates and better neurological scores, yet no significant reduction in infarct size compared to untreated controls (Peinado et al. 2021). Despite these discrepancies, likely due to differences in experimental models, the preponderance of preclinical evidence points to Ngb as a protective protein against stroke (Li et al. 2023).

### Ischemia Induces Ngb Overexpression

Under normal conditions, Ngb is mainly expressed in neurons (Hundahl et al. 2008), but under damage and hypoxic conditions it is upregulated and also expressed in non-neural cells from the NVU (Baez et al. 2016; Li et al. 2014a; Kim et al. 2022). Ngb levels have been reported to peak during the acute phase of stroke in humans, with overexpression being particularly prominent in the nervous cells of the peri-infarct area compared to normal brain tissue and the ischemic core (Jin et al. 2010). Increased Ngb expression following ischemia is even detectable in the serum of both MCAO animal models (Shang et al. 2015) and stroke patients (Jin et al. 2010). Studies examining the potential of circulating Ngb as a clinical biomarker are emerging. Recent reports show that elevated plasma or serum Ngb and glial fibrillary acidic protein (GFAP) levels in acute ischemic stroke patients have been associated with both the severity of neurological symptoms and functional prognosis (Pawluk et al. 2024; Ramli et al. 2024). However, the clinical utility of Ngb as a biomarker remains an active area of investigation; findings regarding the specific time points, stroke subtypes, and the direction of prognostic value remain mixed, requiring further clinical validation before routine application.

The genetic mechanisms underlying Ngb expression under ischemic conditions involve the Ngb gene promoter region, which is regulated by several transcription factors including hypoxia-inducible factor 1α (HIF-1α), serine protease inhibitor factor, early growth response protein 1, nuclear factor NF-kappa-B (NF-κB), and CREB (cAMP response element-binding protein) (Haines et al. 2012; Liu et al. 2012, 2013; Qiu and Chen 2014; Xun et al. 2018). Shang et al. (2015) reported in a rat MCAO model that the number of Ngb-positive hippocampal and cortical cells increased throughout the reperfusion period. They observed increases in Ngb and HIF-1α transcripts and protein levels in the ischemic cortex, peaking at 32 h after MCAO onset, indicating a significant role for HIF-1α in Ngb upregulation following ischemia. The neuroprotective activity of various compounds against stroke has been linked to their ability to induce Ngb expression. Examples include polydatin (Zhang et al. 2022), certain phytoestrogens (Liu et al. 2016), *Prunus cerasoides* extracts (Kim et al. 2021), short-chain fatty acids like cinnamic and valproic acids (Jin et al. 2011), hormones such as estradiol and testosterone (Cabezas et al. 2019), and growth factors like platelet-derived growth factor (Cabezas et al. 2018). Nevertheless, Ngb’s neuroprotective functions against stroke appear effective only when its expression is significantly increased, at least threefold compared to its physiological levels (Fiocchetti et al. 2017). This crucial consideration, independent of enhanced Ngb expression due to an ischemic stimulus, forms the primary rationale for exploring therapeutic interventions (Blanco et al. 2022).

### Overview of the Ngb Neuroprotective Mechanisms

Although the molecular basis of Ngb activity is increasingly understood, fully elucidating how these mechanisms operate within the complex in vivo environment to improve stroke prognosis remains an ongoing challenge. Fundamentally, Ngb’s neuroprotective effects are dictated by its biochemical structure, intracellular availability, and the specific conditions of the ischemic microenvironment. Collectively, these factors dictate how Ngb may alleviate neuroinflammatory damage.

A primary area of interest concerning Ngb under hypoxia stems from its potential role in oxygen transport and storage within neurons. This function may enable neurons to maintain adequate oxygen supply, thereby preserving mitochondrial function, reducing oxidative damage, and enhancing neuronal survival. Studies show that aquatic animals adapted to chronic hypoxia exhibit significantly higher brain Ngb levels (Avivi et al. 2010; Schneuer et al. 2012). This observation, coupled with Ngb’s hypoxia-induced migration towards oxygen sources to facilitate neuronal oxygenation, strongly supports its respiratory role (Li et al. 2023).

However, Ngb’s neuroprotective activities extend beyond oxygen handling, encompassing participation in G-protein signaling, ion homeostasis, antioxidant defense, lipid raft signaling, and gene regulation (Fiocchetti et al. 2017). Specifically, Ngb reversibly binds not only to O_2_ but also to CO and nitric oxide (NO), undergoing conformational changes that control the O_2_/NO ratio and modulate G-protein signalling in hypoxic cells (Brunori and Vallone 2006).

Ngb upregulation under hypoxia mitigates oxidative stress following cerebral ischemia throughout various mechanisms, maintaining the membrane level of sodium/potassium-transporting ATPase subunit beta-1 and preserving Na^+^/K^+^ ATPase β1 activity by reducing reactive oxygen species (ROS)-mediated glutathionylation (Wen et al. 2018). Its physical and functional link with mitochondria further supports its antioxidant role, as seen in findings from hypoxic N2a cell cultures (Li et al. 2023). Ngb also exhibits nitrite reductase activity, influencing nitrite-dependent-NO signalling to protect cells from nitrosative stress (Qiu and Chen 2014). Furthermore, silencing the Ngb gene with siRNA in H_2_O_2_-treated N2a cells led to a loss of mitochondrial membrane potential and increased reactive oxygen species (ROS) levels (Ye et al. 2009).

Ngb also plays a significant role in preventing neuronal death, as under hypoxia its heme group adopts a hexacoordinated structure, facilitating the rapid transfer of electrons from ferrous neuroglobin to ferric cytochrome c. Maintaining non-apoptotic levels of ferrous cytochrome c may prevent the activation cascade of apoptosis (Semenova et al., 2024). Moreover, Ngb’s antiapoptotic function extends beyond direct cytochrome c interaction, involving the inhibition of proapoptotic proteases such as Caspase-3 and Caspase-9 via the PI3K/Akt pathway (Amri et al. 2017). Recent findings by Zhang et al. (2023) indicate that Ngb overexpression after ischemia decreases intracellular Ca^2+^ levels and mitigates mitochondrial dysfunction and endoplasmic reticulum stress, potentially mediated by Ngb’s binding to Synaptotagmin-1. Additionally, Ngb’s redox state may regulate key transcription factors like HIF-1α and nuclear factor erythroid 2–related factor 2 (Nrf2), as well as cytochrome c release, thereby protecting neurons from damage and death during hypoxia (Hota et al. 2012).

Expanding on these molecular interactions, in vivo proteomic analyses utilizing nanoparticle-mediated Ngb delivery have provided a broader perspective on its therapeutic reach (Peinado et al. 2021). These studies revealed that, beyond modulating stress responses and autophagy, Ngb influences the expression of proteins involved in structural repair processes (specifically angiogenesis, dendritogenesis, neuritogenesis, and synaptogenesis) all of which are critical for tissue recovery post-stroke.

These findings suggest that the integrated action of Ngb across these molecular pathways, including its regulation of ROS and NO and influence on transcription factors, is instrumental in determining the functional phenotype of the individual NVU cellular components, a topic which will be detailed in the following sections.

## The Complex Cellular Network of the Stroke Response

Ischemia primarily impacts neurons, which are highly vulnerable to oxygen and glucose deprivation, triggering various types of neuronal death depending on the zone and insult severity (Radak et al. 2017). In the ischemic core, rapid necrosis profoundly disrupts neural signaling (Xing et al. 2012). Conversely, penumbral neurons, while affected, largely remain viable and retain recovery potential (Tuo et al. 2022); when cell death does occur in this region, it typically involves regulated programmed processes such as apoptosis, necroptosis, ferroptosis, parthanatos, and pyroptosis (Duan et al., 2025; Gong et al., 2024).

**Fig. 2:**
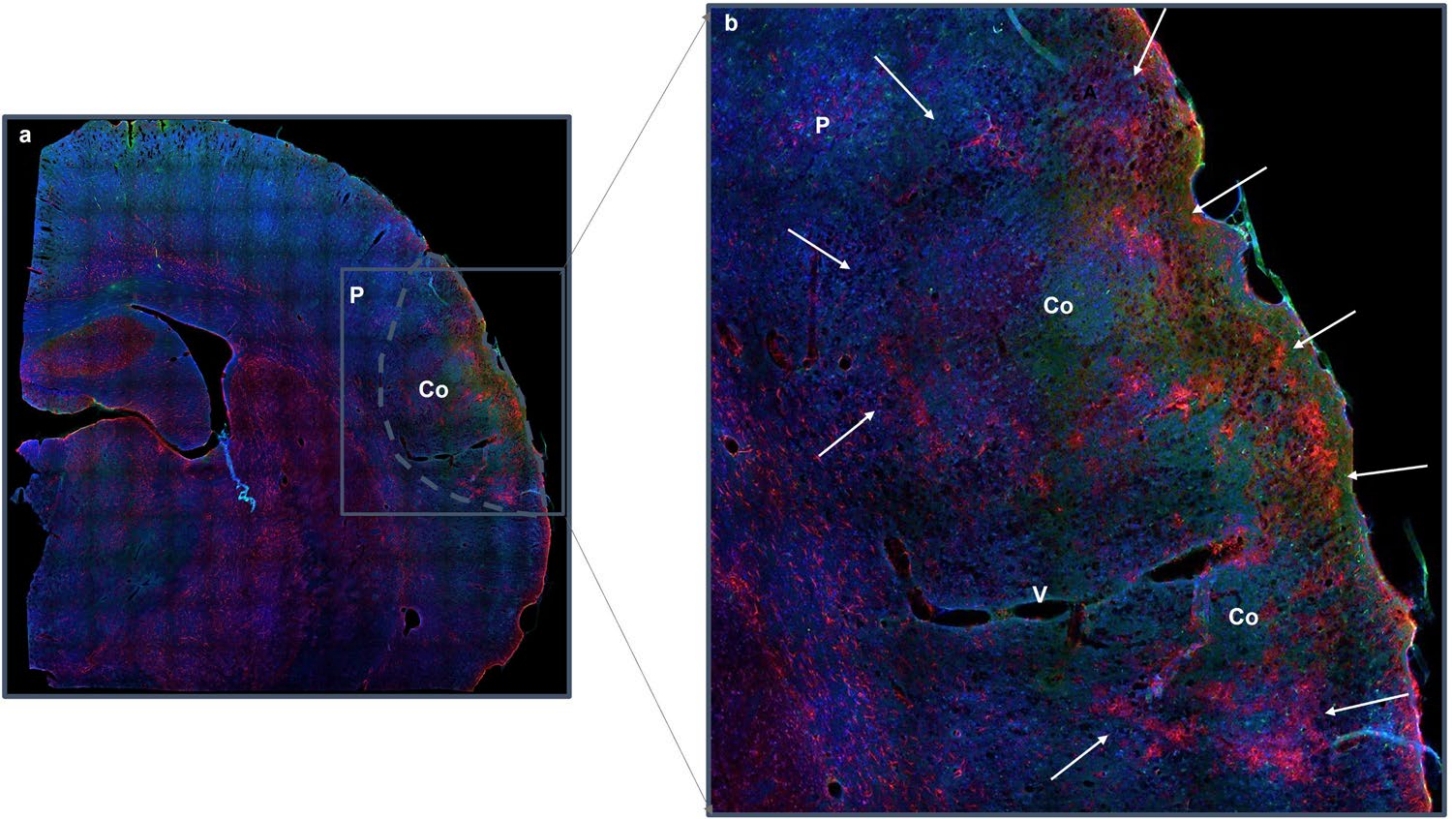
Glial scar formation. **a** Optical section of a brain at the parietal cortex level 24 h after tMCAO illustrating the infarct core (Co) and penumbra (P). **b** A magnified confocal view captures the early organization of proliferating astrocytes (red, GFAP-positive) beginning to form the glial scar boundary (arrows), which delimits the infarct core from the penumbra as have been wide described in the literature and cited in the main text. IBA1-positive microglial cells (green) and cell nuclei (DAPI, blue) are also visualized to provide cellular context. V: vessel. Technical details regarding the acquisition of these illustrative images are published elsewhere (Peralta et al. 2019; Blanco et al. 2020; Peinado et al. 2021)

Within the infarct, neuronal damage triggers reactive gliosis, a process notably affecting microglia and astrocytes. As reported by Liang et al. (2023), this gliosis is not uniform and its intensity varies significantly between the core and the penumbra. This heterogeneity leads to the formation of a glial barrier or scar that delimits these two zones, a process driven by the activation of glial cells undergoing marked morphological and functional transformations. To aid in the conceptualization of these structural changes, Fig. 2 are included to visualize the early stages of glial reactivity. These images illustrate the canonical response to ischemic injury as extensively detailed in the literature (e.g., Sims and Yew 2017; Sofroniew 2009).

This gliotic response involves spatially and temporally dependent structural and functional alterations and is often accompanied by BBB disruption, facilitating immune cell infiltration. Consequently, glial, vascular, and immune cells form an integrated neurovascular network, exchanging factors that exert both detrimental and beneficial effects (Greenhalgh et al. 2020). Furthermore, neuronal damage also impacts distal axons and oligodendrocytes, leading to myelin disorganization and impaired neural communication (Qin et al. 2022).

### Microglia

**Fig. 3:**
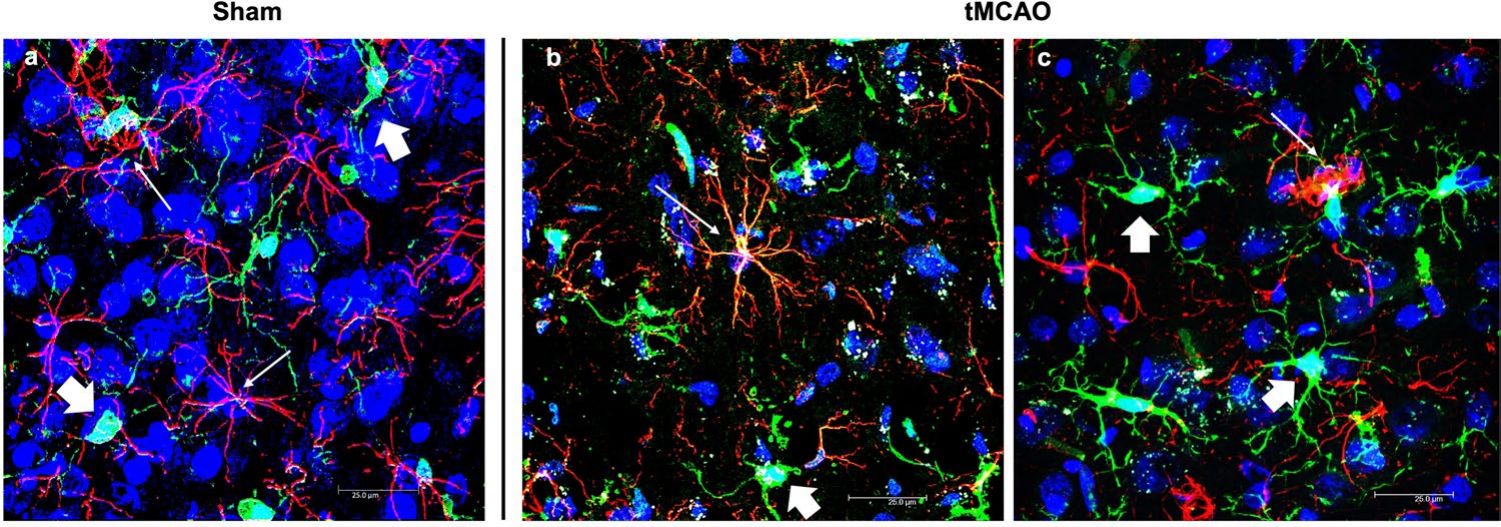
Visualization of the canonical glial activation. Confocal images from the parietal cortex provided to illustrate the early morphological transition of microglia and astrocytes in response to ischemic injury, as described in the literature and cited in the main text. **a** Sham animals display resting astrocytes (GFAP, red; thin arrows) and microglia (IBA1, green; thick arrows), both exhibiting a ramified morphology with fine, long processes. **b**-**c** In response to tMCAO (24 h), both cell types undergo marked activation. Microglia transition to an amoeboid phenotype (retracted processes, enlarged soma), and astrocytes show hypertrophy and increased GFAP expression, indicative of reactive gliosis. Nuclei are stained with DAPI (blue). The technical procedures used for this immunostaining are published elsewhere (Peralta et al. 2019; Blanco et al. 2020; Peinado et al. 2021)

These cells are crucial components in the initial response to stroke. As the central nervous system’s (CNS) resident macrophages, they maintain their population through local proliferation and infiltration of bone-marrow-derived precursors. In their resting state, microglia maintain tissue homeostasis through constant environmental and immune surveillance, displaying a quiescent phenotype with long, thin, and highly branched processes (Xu et al. 2020). Following stroke, microglia transition from their homeostatic ramified state to an amoeboid activated phenotype, a morphological shift widely described in the literature and visually illustrated in Fig. 3.

This activation, or microglial polarization, leads to a wide spectrum of functional states. While historically categorized into the classically activated pro-inflammatory (M1) and the alternatively activated repair-associated (M2) phenotypes, recent single-cell techniques and transcriptomic studies have revealed the broad functional heterogeneity and the continuum of dynamic states exhibited by polarized microglia (Liang et al. 2023).

As reviewed by Jiang et al. (2020), the dynamic phenotypic transition throughout post-stroke stages is influenced by a diverse array of molecular modulators, including transcription factors (such as Nuclear Factor-kappa B [NF-κB] and Signal Transducer and Activator of Transcription [STAT] for pro-inflammatory states, and Nrf2 and Peroxisome Proliferator-Activated Receptor gamma for repair-associated states), key receptors and ion channels (e.g., Toll-Like Receptor 4, sphingosine 1 phosphate receptors, the Voltage-Gated Proton Channel 1, and the Voltage-Gated Potassium Channel 1.3), various gene modulators (like the long non-coding RNA H-19, miRNA-155, miRNA-124), and the TAFA gene family for repair-associated states.

The pro-inflammatory phenotype is characterized by the production of inflammatory cytokines like Tumor Necrosis Factor -α (TNF- α), interleukin (IL)−1β, and, and Interleukin-6 (IL-6), along with ROS and NO. Excessive NO synthesis, largely via the Ca^2+^-dependent inducible nitric oxide synthase (iNOS) enzyme—traditionally considered a hallmark marker of this state—causes nitrosative stress and neuronal damage (Rodrigo et al. 2001; Hu et al. 2012). The release of these pro-inflammatory molecules, along with factors like metalloproteinases (MMPs), contributes to neuronal damage and BBB disruption, exacerbating the inflammatory response (Amantea et al. 2015).

Conversely, the repair-associated phenotype suppresses inflammation by releasing various anti-inflammatory cytokines, including IL-4, IL-10, and Transforming Growth Factor β (TGF-β), associated with signaling pathways linked to CREB and Nrf2 (Liang et al. 2023). Repair-associated microglia express arginase-1 (ARG1), an enzyme that competes with nitric oxide synthase for intracellular arginine, preventing excessive NO synthesis and damage. Additionally, a neuroprotective signaling axis, the STAT6/ARG1, has been identified in this restorative microglial phenotype; it is involved in phagocytic clearance of dying and dead cells, a necessary mechanism for restoring brain homeostasis after stroke (Cai et al. 2019). These pathways promote repair and regeneration, enhancing phagocytosis, removing cellular debris, and contributing to tissue clearance (Lan et al. 2017; Jiang et al. 2020).

Various markers are used to identify microglia and their activation states. These include Cluster of Differentiation molecule 11b, Major Histocompatibility Complex class II, CX3C Motif Chemokine Receptor 1, and the microglia-specific Transmembrane Protein 119, alongside widely recognized markers such as IBA1, iNOS, and ARG1 (Jiang et al. 2020). Among these, IBA1 is the most widely used marker for studying the morphological changes of the entire microglial population (including bone-marrow precursors). IBA1 binds Ca^2+^ and interacts with actin, being involved in cytoskeletal reorganization, migration, and phagocytosis (Jiang et al. 2020). In resting microglia, IBA1 is primarily localized in the cytosol, but upon activation, it undergoes upregulation, redistributing toward the plasma membrane and cellular processes, paralleling the morphological and functional changes of activated phenotypes.

Regarding the temporal evolution of microglia after stroke, it has been hypothesized that the balance between phenotypes (traditionally termed the M1/M2 balance) might first exhibit a neuroprotective (repair-associated) profile in early stages before transitioning to a pro-inflammatory (deleterious) state in later stages (Liang et al. 2023). However, the intricate variability of ischemic injury evolution across different reperfusion times and infarct zones, a complexity already highlighted in seminal works (Iadecola and Anrather 2011), continues to present a challenge that requires further elucidation to optimize therapeutic timing.

### Astrocytes

These cells work in concert with microglia to maintain the delicate post-stroke balance between damage and repair. In their resting state, astrocytes are responsible for preserving BBB integrity, providing metabolic support to neurons—including neurotransmitter uptake and release—and regulating ion homeostasis (Xu et al. 2020). Following stroke, astrocytes undergo rapid activation characterized by cell body hypertrophy, proliferation, and migration. This morphological transition is visually captured in Fig. 3, which illustrates the concurrent activation of both astrocytes and microglia in the ischemic tissue.

Activated astrocytes experience diverse, time-dependent changes in cell surface receptors, ion channels, ion transporters, and GTPases, triggering signaling pathways critical for post-stroke evolution. These cells release antioxidants, free radical scavengers, and neurotrophic factors that reduce neuronal damage. Moreover, their moderate proliferation, initiating in the early post-stroke stages and modulated by microglia, contributes to the formation of the glial scar, a structural barrier that prevents the spread of the ischemic lesion (Chen et al. 2024).

A hallmark of this activation is the upregulation of GFAP (Chen et al. 2024; Gorina et al. 2022). GFAP is a specific intermediate cytoskeletal filament that ensures cell stability and regulates astrocyte morphology and function. Post-stroke, GFAP expression is spatiotemporally regulated by molecules released from surrounding cells and by astrocytes themselves (Norden et al. 2016). These regulatory molecules include pro-inflammatory cytokines (e.g., TNF-α, IL-1β, IFN-γ, TGF-β), Fibroblast Growth Factor, neurotransmitters (e.g., glutamate), and transcription factors (NF-κB, activator protein-1, CREB, STATs), which bind to the GFAP promoter region to enhance gene transcription. Epigenetic modifications, such as histone acetylation and DNA methylation, can also alter GFAP gene accessibility, further influencing its expression (Qin et al. 2022). Given that astrocytes differentiate into complex and heterogeneous subtypes post-stroke, GFAP provides a reliable tool for studying this evolution (Pawluk et al. 2024).

Activated astrocytes represent a continuum of dynamic and heterogeneous responses. While historically categorized into neurotoxic (A1) and neuroprotective (A2) phenotypes—mirroring the microglial classification—recent transcriptomic studies support a more complex functional landscape (Liddelow and Barres 2017). Consequently, contemporary research prioritizes functional descriptors. The neurotoxic/inflammatory phenotype is associated with neuronal damage and poor functional recovery. This state is induced by factors released from pro-inflammatory microglia (e.g., IL-1α, TNF-α) triggering signaling pathways involving NF-κB. Furthermore, this phenotype releases pro-inflammatory cytokines, such as IL-1β, and the neurotoxic Complement component 3 d, both contributing to neuronal death. Distinct from this is the scar-forming phenotype, characterized by proliferation and increased GFAP expression to limit lesion spread (Chen et al. 2024). Conversely, the neuroprotective/repair-associated phenotype secretes factors like Brain-Derived Neurotrophic Factor and Glial Cell Line-Derived Neurotrophic Factor, which promote neuronal repair and regeneration (Baez et al. 2016; Liu et al. 2020). Regulating the balance between these functional states offers a valuable therapeutic target for reducing neuroinflammation and promoting recovery.

A significant aspect of post-stroke glial activation is the bidirectional interaction of astrocytes with other glial cells and infiltrating peripheral immune cells. Astrocytes may form a syncytium with other glial cells via gap junctions to facilitate synchronous post-stroke responses (Yu et al. 2024). Microglia and astrocytes bidirectionally exchange signals and influence each other at various stages of the post-ischemic period. Initially, microglia induce astrocytes to form the glial scar to limit inflammation; in later stages, microglia initiate mechanisms to remodel or remove the scar, as its persistence can hinder recovery. This cell-cell interaction involves astrocytic Connexin 43 (CX43) hemichannels (Liang et al. 2023) and the release of microglial extracellular vesicles or exosomes containing modulating molecules (Gao et al. 2024). These exchanges, including pro- and anti-inflammatory cytokines, growth factors, and microRNAs (miRNAs), regulate astrocyte gene expression and drive phenotypic and functional changes (Song et al. 2023), potentially unlocking the neurogenic capacity of astrocytes to convert into functional neurons (Guo et al. 2023).

### Oligodendrocytes

These cells are highly vulnerable to ischemic damage post-stroke (Cheng et al. 2024), a susceptibility that significantly impacts their function and contributes to neurological deficits. As the producers of myelin essential for efficient nerve signal transmission, they are highly sensitive to excitotoxicity, oxidative stress, and inflammatory mediators. Oligodendrocytes exhibit damage and morphological changes within 3 h post-ischemia, compromising myelin sheath integrity; this, coupled with axonal disorganization resulting from neuronal death, impairs neuronal communication and leads to long-term sensory, motor, and cognitive impairments (Yu et al. 2024).

Activated microglia and astrocytes regulate post-ischemic oligodendrocyte behavior. During acute and subacute stages, the microglial pro-inflammatory phenotype induces oligodendrocyte damage and apoptosis in the white matter. Conversely, the repair-associated microglial phenotype promotes remyelination. Furthermore, Vascular Endothelial Growth Factor-C, released by activated cells, stimulates Oligodendrocyte Progenitor Cell (OPC) proliferation post-ischemia (Xu et al. 2020).

The post-ischemic astrocytic syncytial network also influences oligodendrocyte evolution. The astrocytic hemichannel protein CX43, combined with oligodendrocyte-expressed Connexin 47 (CX47), forms hybrid gap junctions promoting calcium ion and glucose interchange (Niu et al. 2016). Moreover, direct communication allows astrocytes to transfer exosomes containing Laminin Subunit Beta 2 (LAMB2) to oligodendrocytes. LAMB2 not only structurally contributes to the vascular basal lamina but also induces adult OPC differentiation into new functional oligodendrocytes, aiding in the repair of injured white matter (Jiang et al. 2019; Cheng et al. 2022). Reactive astrocytes also recruit OPCs and contribute to repair by clearing myelin debris (Jia et al. 2019). OPC precursor stem cells, identified as Neural/Glial Antigen 2 (NG2)-expressing cells, comprise 5–8% of all adult CNS cells. While they primarily develop into oligodendrocytes, the characteristic NG2 marker has been found colocalized with astrocyte and neuronal markers, indicating their potential to differentiate towards astrocytes and even neurons (Janeckova et al. 2024). Thus, the neurogenic potential of NG2 cells makes them promising targets for post-stroke therapeutic strategies.

### Pericytes and Other Cells from NVU

Endothelial cells and pericytes play essential roles in maintaining the BBB alongside neurons and glia (Candelario-Jalil et al. 2022). Pericytes are critical to the NVU response to ischemia. These perivascular mural cells, embedded within the basal lamina surrounding endothelial cells, regulate vascular functions including BBB formation and maintenance, vessel maturation, blood flow, and immune cell trafficking. Roth et al. (2025) reported that approximately 30% of pericytes die within the first hour post-ischemia, while many detach from the vascular bed, and about 50% express diverse activation markers. Ischemic activated pericytes may secrete pro-inflammatory factors, inducing microglial activation (Matsumoto et al. 2014), but also contribute to repair (Kim et al. 2022). Furthermore, evidence suggests pericytes can differentiate into neural and vascular lineage cells after ischemia, playing vital roles in post-ischemic recovery (Janeckova et al. 2024).

Blood flow disruption significantly impacts these vascular components. Endothelial cells undergo rapid changes in tight and adherens junctions and membrane transporters. Simultaneously, the detachment and activation of pericytes contribute to the breakdown of the BBB. These changes are regulated by the intricate interplay among activated astrocytes, microglia, the pericytes themselves, and infiltrating blood cells, all of which critically influence BBB evolution during reperfusion (Jiang et al. 2019). As previously described, activated NVU cells release chemokines, cytokines, and MMPs. These neuroinflammatory molecules induce pathological BBB breakdown, allowing the influx of immune cells, inflammatory substances, fluid, and proteins into the CNS. This influx causes inflammation and edema (increasing intracranial pressure), contributes to neuronal damage, and accelerates cell death. Conversely, anti-inflammatory signals may protect the BBB, fostering recovery (Lu & Wen, 2024).

The role of peripheral blood cells entering the brain parenchyma post-stroke is noteworthy. The upregulation of endothelial adhesion molecules facilitates the infiltration of various peripheral immune cells. Among these, neutrophils release additional inflammatory cytokines and chemokines, further activating glial cells (Yang et al. 2019); they also produce intravascular and intraparenchymal neutrophil extracellular traps that reduce neovascularization and exacerbate BBB damage (Kang et al. 2020). Other significantly involved infiltrating cells, besides monocytes (microglial precursors), are T regulatory cells. These cells appear to influence BBB integrity during acute stroke, though they may exert a neuroprotective role in the subsequent chronic post-stroke phase (Liesz and Kleinschnitz 2016).

Given the complex interplay of NVU cells and the centrality of BBB integrity in stroke outcome, a key area of investigation is how neuroprotective agents regulate these cellular interactions. Studies suggest Ngb modulates the activation and crosstalk of various NVU cell types, including glial cells and pericytes, during post-stroke events. Consistent with this, elevated Ngb levels in these cells appear to exert protective effects by preserving endothelial and pericyte integrity, limiting secondary injury, promoting repair, and improving post-stroke recovery (Blanco et al. 2022).

## Neuroglobin in the Interplay Within the Neurovascular Unit

As detailed previously, ischemia induces Ngb upregulation across NVU cells as an endogenous protective response; however, this physiological increase is often insufficient to fully counteract ischemic damage (Fiocchetti et al. 2017). Consequently, therapeutic strategies enhancing Ngb levels offer a promising avenue for neuroprotection. While earlier systemic delivery attempts faced limitations due to the protein’s inability to cross the blood-brain barrier (BBB) (Cai et al. 2011; Fiocchetti et al. 2017), the development of Ngb-linked nanoparticles (Ngb-NPs) has successfully overcome this hurdle, achieving effective delivery to NVU cells (Peralta et al. 2019; Blanco et al. 2020, 2022; Peinado et al. 2021). The following sections address how this interplay between endogenous and potentially therapeutic Ngb modulates glial activation and cellular communication to foster a neuroprotective environment.

### Neuroglobin and Glial Activation

The protective mechanisms triggered by Ngb upregulation emerge from diverse experimental models. A crucial finding in a rodent tMCAO model is that Ngb upregulation preferentially occurs in the penumbra rather than the ischemic core (Kim et al. 2022), suggesting a targeted response in regions where tissue is still viable.

In microglial cells, Ngb expression follows a temporal pattern, with mRNA peaking at 6 h and protein levels at 24 h post-injury (Li et al. 2014b). Ngb modulates activation by suppressing pro-inflammatory pathways (e.g., NF-κB and Janus kinase/STAT) and reducing inflammatory cytokine release, thereby shifting the balance toward a repair-associated phenotype (Xun et al. 2018). For instance, the anti-neuroinflammatory effects of CO in microglia are Ngb-dependent, involving the reduction of iNOS and TNF-α and the upregulation of IL-10 (Días-Pedroso et al., (Dias-Pedroso, et al., 2022)). Similarly, tibolone’s ability to preserve mitochondrial membrane potential in microglia has been proposed to be mediated by Ngb upregulation (Avila-Rodriguez et al. 2016). Furthermore, Wang et al. (2022) highlighted that the protective function of the repair-associated microglial phenotype is linked to the co-expression of Ngb and Heme Oxygenase-1.

In astrocytes, stroke-induced polarization by factors like HIF-1α and NF-κB also drives significant Ngb upregulation (Haines et al. 2012; Cabezas et al. 2018). This increase protects astrocytes from ischemic injury, reinforcing their ability to regulate neuroinflammation and ion homeostasis (Baez et al. 2016). This intrinsic protection is underscored by Ngb’s anti-apoptotic function via its interaction with cytochrome c (De Marinis et al. 2013). Interestingly, activated astrocytes may serve as a reservoir, storing Ngb for potential transfer to surviving neurons via exosomes, representing a key mechanism of bystander protection (Venturini et al. 2019).

Hormonal regulation introduces further complexity to astrocytic Ngb expression. Estradiol can activate Ngb expression directly via genomic pathways (binding nuclear receptors ERα and ERβ with transcription factors such as Sp1, activator protein 1, and NF-κB) or indirectly via non-genomic pathways involving membrane-bound receptors (GPER1) and kinase signaling (e.g., p38/MAPK) (De Marinis et al. 2010). As reviewed by Barreto et al. (2021), while ERβ regulates basal expression, maintaining Ngb upregulation under inflammatory stress requires synergy between ERα and ERβ. Testosterone also induces astrocytic Ngb expression under glucose deprivation (Cabezas et al. 2018), indicating that Ngb regulation is highly context-dependent.

Ngb also modulates the activation of pericytes. Elevated Ngb levels in these cells limit oxidative stress and apoptosis, essential for maintaining pericyte viability and BBB integrity. In post-stroke stages, pericytes in the penumbra undergo significant Ngb upregulation, specifically in those expressing Platelet-Derived Growth Factor Receptor beta (PDGFRβ) (Kim et al. 2022). Upon ligand binding, PDGFRβ induces pericyte proliferation and survival. Notably, Ngb-positive PDGFRβ pericytes remain associated with endothelial cells, reducing BBB leakage, unlike other pericyte subpopulations that detach and contribute to barrier breakdown (Cabezas et al. 2018).

Beyond cell survival, Ngb has been linked to neurogenesis and repair. Pericytes may differentiate into other NVU cell types post-ischemia, a potential modulated by Ngb via the Wnt signaling pathway (Yu et al. 2018), a finding supported by our proteomic analyses using Ngb-NPs (Peinado et al. 2021). Additionally, Ngb promotes neurite outgrowth and axon regeneration through the PI3K/Akt pathway (Li et al. 2014b)d -Ngb-p38-growth associated protein 43 signaling during reperfusion (Xiong et al. 2018).

Collectively, these findings highlight the self-defensive role of Ngb upregulation across NVU components. To better understand this role, and considering the significant consequences of BBB disruption, it is crucial to delve deeper into the crosstalk among all Ngb-expressing cells. This is particularly relevant when designing systemic therapies, as any effective neuroprotective agent must not only cross the BBB but also orchestrate a synchronized response across this integrated cellular network.

### Therapeutic Neuroglobin

**Fig. 4:**
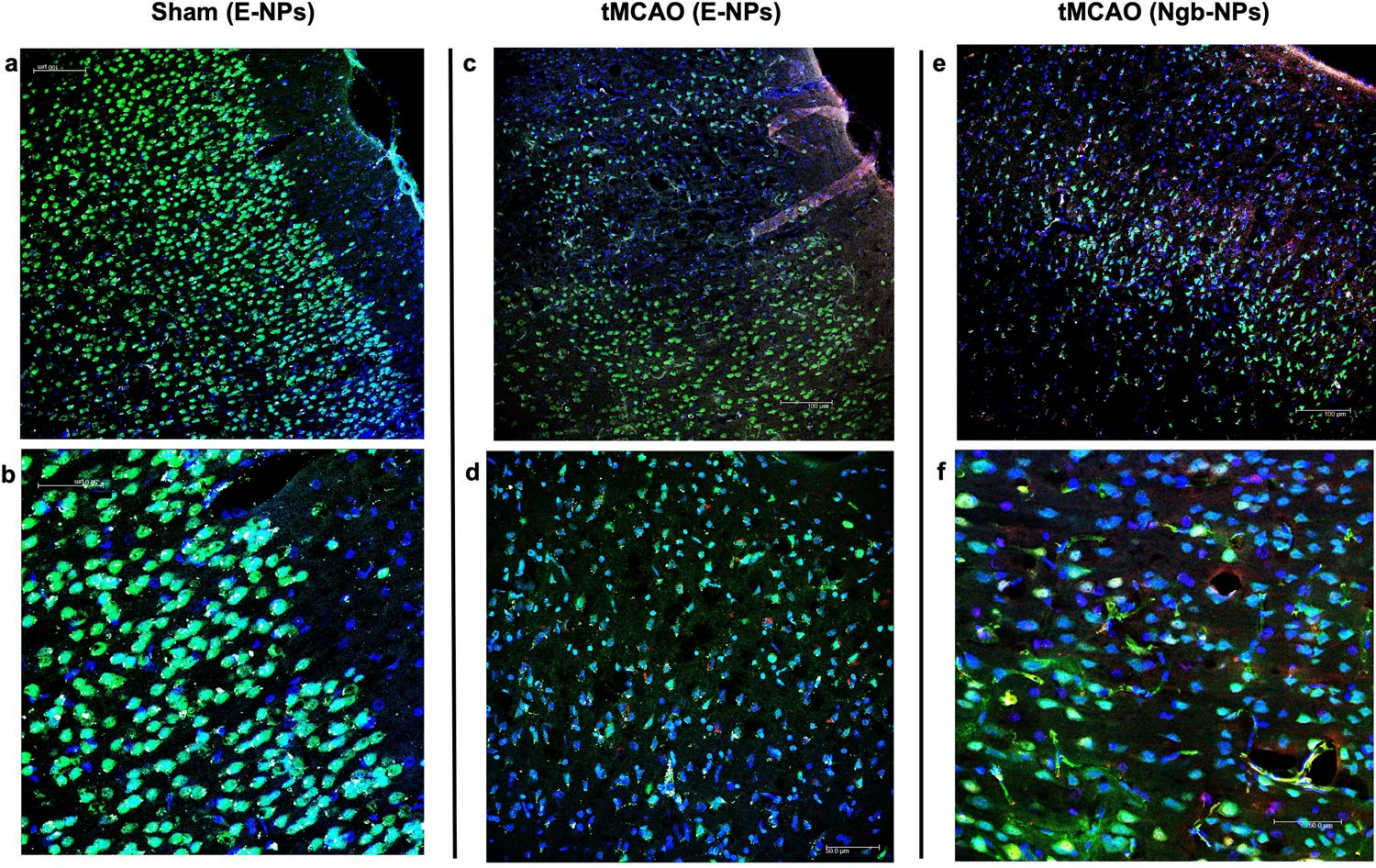
Visualization of blood-brain barrier penetration by Ngb-linked nanoparticles. Confocal images of the parietal cortex provided to illustrate the effective delivery of systemic nanoparticles (NPs) to the brain parenchyma 24 h post-surgery, as previously characterized (Peralta et al. 2019; Blanco et al. 2020). The panels illustrate the distribution of NPs in (**a**, **b**) Sham animals, (**c**, **d**) non-treated tMCAO animals, and (**e**-**f**) Ngb-NP treated animals. **e**-**f** In the treated group, the presence of Ngb-NPs is evidenced by the overlapping of NPs (rhodamine, pseudocolored white) and Ngb (red). Neurons are stained with NeuN (green) and nuclei with DAPI (blue). Technical details regarding the acquisition of these images are published elsewhere (Peralta et al. 2019; Blanco et al. 2020; Peinado et al. 2021)

To address the challenge of BBB penetration, therapies based on systemic (Ngb-NPs have emerged as a promising strategy for improving ischemic outcomes (Blanco et al. 2022). Validating this approach, previous studies from our group confirmed that systemic Ngb-NPs successfully cross the BBB, reaching ischemic neurons and other NVU cells as early as 2 h post-injection and remaining detectable 24 h later, as visually recapitulated in Fig. 4 (Peralta et al. 2019; Blanco et al. 2020, 2022). Furthermore, data from Ngb-treated tMCAO animals indicated that this therapy not only induced proteomic changes associated with regenerative processes but also significantly improved mortality rates and behavioral scores at 24 h post-ischemia (Peinado et al. 2021).

Despite these encouraging preclinical results, translating Ngb therapy requires addressing critical challenges, such as optimizing nanoparticle safety and defining the precise therapeutic window. Although the 24-hour timeframe represents an acute stage requiring longer-term monitoring, it is a critical window for initiating NVU repair. The administration of exogenous Ngb could modulate the protective responses of all NVU components, including glia and the vasculature, offering a multimodal approach to neuroprotection.

Specifically, the influence of exogenous Ngb on astrocytic and microglial responses in the acute phase is of paramount importance. In astrocytes, this involves mitigating vasogenic edema, reducing acute inflammation, supporting BBB integrity, and promoting tissue regeneration via endothelial-stabilizing factors (Sofroniew and Vinters 2010). Crucially, Ngb modulation may help balance glial scar formation to facilitate axonal regeneration and functional recovery (Fawcett 1999; Sofroniew 2009). This dynamic suggests that Ngb could facilitate the microglial transition by inhibiting pro-inflammatory phenotypes (Xun et al. 2018; Dias-Pedroso, et al., 2022); however, elucidating the specific molecular mechanisms driving these shifts requires further investigation.

Ngb therapy also offers direct benefits to vascular components, given its role in preserving pericyte viability and BBB integrity (Kim et al. 2022). Moreover, under hypoxic conditions, the ability of Ngb to act as a nitrite reductase (converting nitrite to NO as an alternative to oxygen-dependent NOS activity) is a relevant therapeutic mechanism (Tiso et al. 2011). This Ngb-derived NO is vital for vasodilation and increasing blood flow to oxygen-deprived tissues (Brunori et al. 2005), potentially complementing NOS-produced NO to counteract hypoxic vasoconstriction.

## Data Availability

No datasets were generated or analysed during the current study.
